# Diverse RNA Viruses Discovered in Three Parasitoid Wasps of the Rice Weevil *Sitophilus oryzae*

**DOI:** 10.1128/mSphere.00331-21

**Published:** 2021-05-05

**Authors:** Fei Wang, Bo Yuan, Shan Xiao, Jiao Zhang, Wenxi Jia, Qi Fang, Fang Wang, Qisheng Song, Gongyin Ye

**Affiliations:** a State Key Laboratory of Rice Biology, Ministry of Agriculture and Rural Affairs Key Laboratory of Molecular Biology of Crop Pathogens and Insects, Institute of Insect Sciences, Zhejiang University, Hangzhou, China; b Division of Plant Sciences, College of Agriculture, Food and Natural Resources, University of Missouri, Columbia, Missouri, USA; Nanjing Normal University

**Keywords:** RNA viruses, parasitoid wasp, rice weevil, *Picornavirales*, *Mononegavirales*, RdRp

## Abstract

In this study, many virus-like fragments were obtained from transcriptomes of three wasp species, including Anisopteromalus calandrae (8), Lariophagus distinguendus (3), and Theocolax elegans (18), which can parasitize and control rice weevil *Sitophilus oryzae*, a serious insect pest of farm-stored grains. By further bioinformatic analysis and sequencing, we identified six novel RNA viruses with complete genomes and named them WWPSRV-1, WWPSRV-2, AcPSRV-1, AcNSRV-1, AcNSRV-2, and LdNSRV-1. PCR-based detection revealed that WWPSRV-1 and WWPSRV-2 had the possibility of interspecies virus transmission, especially WWPSRV-2, which was also present in the rice weevil adults. Phylogenetically, three out of these six viruses appeared to be members of order *Picornavirales*: WWPSRV-1 belonged to unassigned virus families of this order, whereas WWPSRV-2 and AcPSRV-1 belonged to families *Iflaviridae* and *Dicistroviridae*, respectively. The conserved picornavirus-typical domains helicase, protease, and RNA-dependent RNA polymerase could be found in the nonstructural protein encoded by the three viruses, whose genomes consisted of the different numbers of open reading frames (ORFs). The other three RNA viruses could be classified to order *Mononegavirales*: AcNSRV-1 and AcNSRV-2 belonged to family *Lispiviridae*, whereas LdNSRV-1 belonged to a big family *Rhabdoviridae*. The genomes of the three viruses contained at least five ORFs, encoding deduced proteins in the following order: 3′-N-P-M-G-L-5′. All the ORFs were separated by conserved intergenic sequences which likely regulated the transcription termination and initiation. Our findings enhance the understanding of RNA viruses in weevil wasps and set the foundation for the future study of the association among weevils, weevil wasps, and RNA viruses.

**IMPORTANCE** The enormous diversity of RNA viruses in insects is continuously validated. Parasitoid wasps, as biocontrol insects which are widely used against insect pests in agroecosystems, may also carry many “good” RNA viruses. Some RNA viruses in parasitoid wasps have been reported to affect the host wasps or the wasps’ host. Here, six novel RNA viruses with complete genomes were identified in three parasitoid wasps of the rice weevil. One of these viruses was also detected in the rice weevil adults. Phylogenetically, WWPSRV-1 was the first unambiguous detection of Nora-like virus in insect parasitoids. WWPSRV-2 and AcPSRV-1 belong to families *Iflaviridae* and *Dicistroviridae*, some viruses of which can result in lethal infections in silkworms and honeybees. The other three RNA viruses belong to order *Mononegavirales*, which comprises many well-known insect-associated viruses.

## Abstract (precis)

The enormous diversity of RNA viruses in insects is continuously validated. Parasitoid wasps, as biocontrol insects which are widely used against insect pests in agroecosystems, may also carry many “good” RNA viruses.

## INTRODUCTION

RNA viruses are increasingly discovered in invertebrate species which display enormous diversity and can be generally divided into positive-, negative-, or double-stranded RNA viruses (1–3). Species abundance and diversity of RNA viruses also exist in insects, the largest group in invertebrates (4–6). Nowadays, the application of metagenomics accelerates the studies of viral identification, persistence, spread, and interaction with both their insect hosts and other microbes. For example, using metagenomic analysis, seven novel positive-strand RNA viruses, belonging to the families *Dicistroviridae*, *Parvoviridae*, and *Circoviridae*, are identified in an important stingless bee Melipona quadrifasciat*a* which have the largest copy numbers in unhealthy bees and may be pathogenic (7). Eight novel RNA viruses, including four positive-strand RNA viruses, two negative-strand RNA viruses, and two double-stranded RNA, are discovered in the high-throughput sequencing data of an important invasive agricultural insect pest, the oriental fruit fly *Bactrocera dorsalis* (8). Through a metatranscriptomics analysis, many new RNA virus species are discovered in different mosquito populations, including many that affect human health. This indicates that the diversity of RNA viruses is affected by the host mosquito taxon and genetic background (9–11). With understanding of mosquito viromes, the prevention and control of pathogenic arboviruses will be more easily manipulated.

Parasitoid wasps (Hymenoptera: Apocrita) are widely used as biocontrol agents against insect (Arthropoda: Hexapoda: Insecta) pests in agroecosystems. They are frequently associated with viruses or virion-like particles (VLPs). In the past decade, RNA viruses have been reported in some parasitoid wasps, as well as in virus-host interactions. Several viruses have been found to affect the host wasps or the wasps’ host. For example, Pteromalus puparum negative-strand RNA virus 1, an artovirus found in Pteromalus puparum wasp which preys on butterflies of several species, increases the longevity of the wasp while it reduces female offspring numbers (12).

The rice weevil, Sitophilus oryzae (Coleoptera: Curculionidae), a serious insect pest of farm-stored grains, is prevalent mainly in the south and the center of China (13). It could cause significant losses in the grain weight and reduce nutritive value (14). Three hymenopteran Pteromalidae parasitoids, including *Anisopteromalus calandrae*, *Lariophagus distinguendus*, and *Theocolax* (*Choetospila*) *elegans*, which can parasitize the rice weevil larvae in nature, have great potential in biological control of S. oryzae (15, 16). To better understand the relationship between these parasitoid wasps and their hosts, we sequenced the transcriptomes of the three wasps. Six novel viruses were discovered in wasps A. calandrae, L. distinguendus, and T. elegans, named weevil wasp positive-strand RNA virus 1 (WWPSRV-1), weevil wasp positive-strand RNA virus 2 (WWPSRV-2), Anisopteromalus calandrae positive-strand RNA virus 1 (AcPSRV-1), Anisopteromalus calandrae negative-strand RNA virus 1 (AcNSRV-1), Anisopteromalus calandrae negative-strand RNA virus 2 (AcNSRV-2), and Lariophagus distinguendus negative-strand RNA virus 1 (LdNSRV-1). Moreover, we analyzed the genome organization and phylogeny of these viruses. Our results revealed that WWPSRV-1, WWPSRV-2, and AcPSRV-1 were related to picornaviruses and AcNSRV-1, AcNSRV-2, and LdNSRV-1 were related to mononegaviruses.

## RESULTS

### Virus-like contigs obtained from the transcriptomes of three parasitoid wasps.

Through transcriptome analysis, 29 virus-like fragments were obtained in three weevil wasp species, including *A. calandrae* (8), *L. distinguendus* (3), and *T. elegans* (18) (Table S3). The majority of small contigs were in *T. elegans*. Based on BLASTX results, these fragments were classified into one of two main groups: positive-strand RNA virus (11) or negative-strand RNA virus (18) (Table S3). All three wasps had these two types of virus-like sequences (Table 1 and Table S3).

**TABLE 1:** The distribution of the viruses in the three different wasps’ transcriptomes^*a*^

Virus	Abbreviation	Length (bp)	*Anisopteromalus calandrae*	*Lariophagus distinguendus*	*Theocolax elegans*
Weevil wasp positive-strand RNA virus 1	WWPSRV-1	12,332	+	+	+
Weevil wasp positive-strand RNA virus 2	WWPSRV-2	9,409	+	+	+
Anisopteromalus calandrae positive-strand RNA virus 1	AcPSRV-1	7,558	+	−	−
Anisopteromalus calandrae negative-strand RNA virus 1	AcNSRV-1	12,702	+	−	−
Anisopteromalus calandrae negative-strand RNA virus 2	AcNSRV-2	12,118	+	−	−
Lariophagus distinguendus negative-strand RNA virus 1	LdNSRV-1	10,832	−	+	−

a +, exists; −, does not exist.

10.1128/mSphere.00331-21.9 TABLE S3 Virus-like contigs identified in three different wasps’ transcriptomes. AC, *Anisopteromalus calandrae*; LD, *Lariophagus distinguendus*; and TE, *Theocolax elegans*. Download Table S3, XLSX file, 0.01 MB. Copyright © 2021 Wang et al. 2021 Wang et al. https://creativecommons.org/licenses/by/4.0/ This content is distributed under the terms of the Creative Commons Attribution 4.0 International license.

### WWPSRV-1, WWPSRV-2, and AcPSRV-1 were related to positive-strand RNA virus.

**FIG 1:**
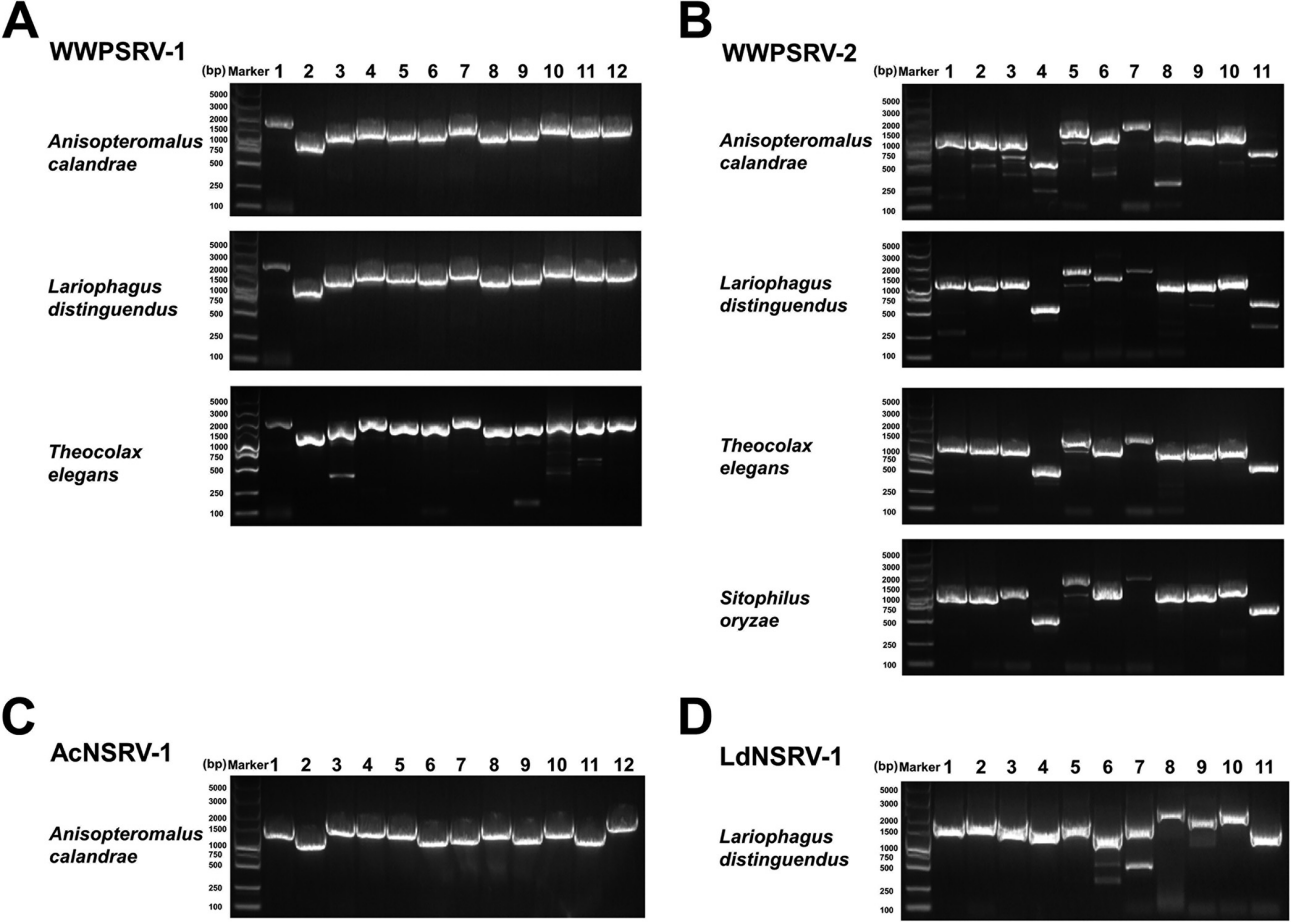
PCR confirmation of WWPSRV-1 (A), WWPSRV-2 (B), AcNSRV-1 (C), and LdNSRV-1 (D) in the parasitoid wasps *Anisopteromalus calandrae*, *Lariophagus distinguendus*, and *Theocolax elegans* and their host *Sitophilus oryzae* adults. The primers used to confirm viral genome sequence are shown in the Table S1.

**FIG 2:**
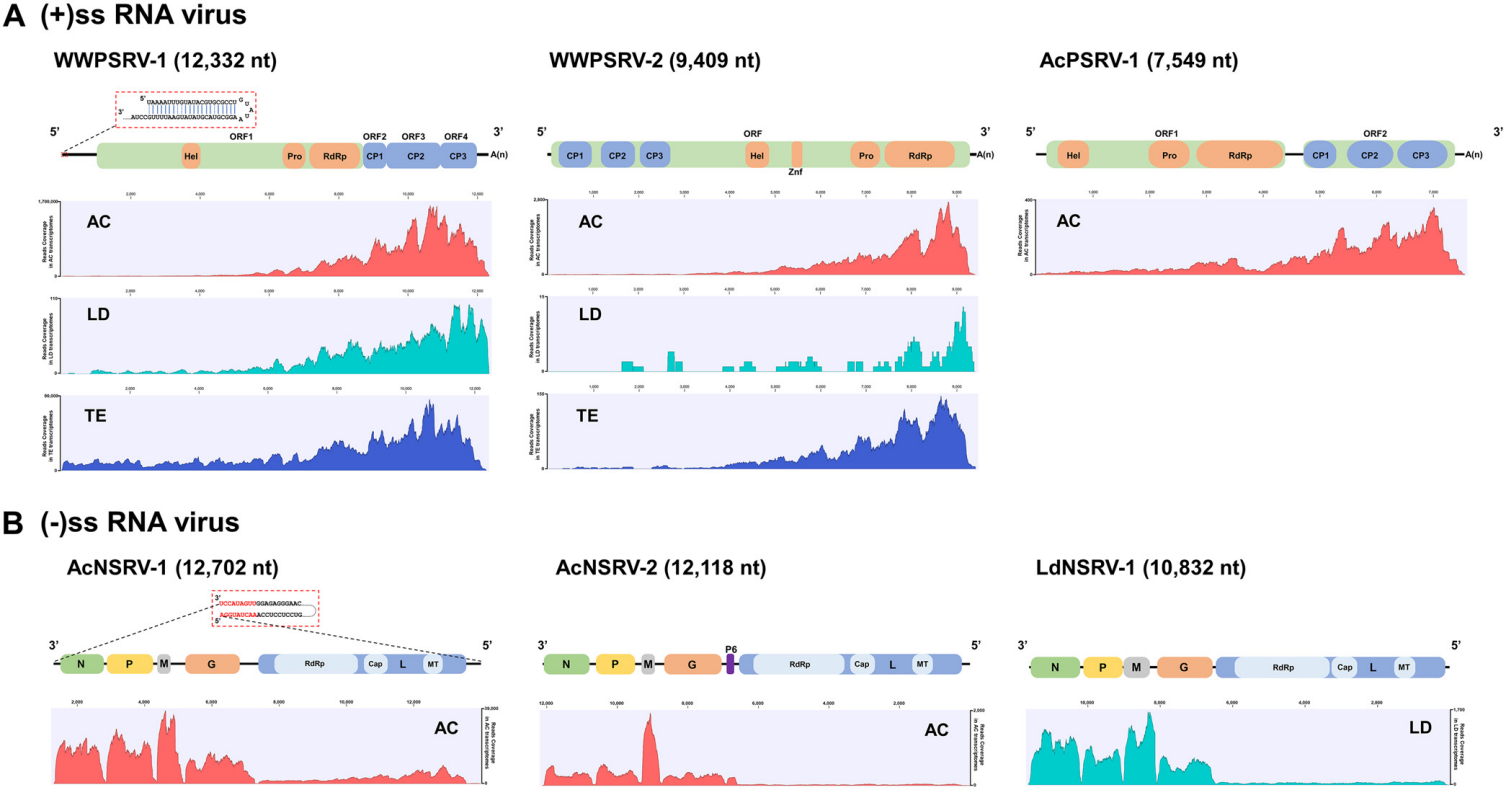
Schematic presentation of the genome of RNA viruses discovered in the *Anisopteromalus calandrae* (AC), *Lariophagus distinguendus* (LD), and *Theocolax elegans* (TE) transcriptomes. (A) Schematic presentation of the genomic organization of positive-strand RNA viruses (WWPSRV-1, WWPSRV-2, and AcPSRV-1). Boxes indicate the position and length of each ORF arranged in the 5′–3′ orientation. In the boxes, the positions of the putative structural proteins (capsid protein, CP 1 to 3) and the nonstructural proteins (helicase, Hel; protease, Pro; RNA-dependent RNA polymerase, RdRp) are shown. There is a zinc finger (Znf) domain in the WWPSRV-2 genome. (B) Schematic presentation of the genomic organization of negative-strand RNA viruses (AcNSRV-1, AcNSRV-2, and LdNSRV-1). Boxes indicate the position and length of each ORF (N, P, M, G, and L genes) arranged in the 3′–5′ negative sense. P6 is found only in the AcNSRV-2 genome. In the L genes, the positions of the putative RdRp, mRNA-capping (Cap), and methyltransferase (MT) domains are shown. RNA-seq mapping for AC (red), LD (green), and TE (blue) showed fluctuating read distributions on viral genomic RNA.

**(i) WWPSRV-1.** Three large contigs obtained from the wasps’ transcriptomes (AC_Contig_1, LD_Contig_1, and TE_Contig_1) (Text S1) were highly related to Nasonia vitripennis virus and could be assembled into one big virus-like contig (12,325 bp), named WWPSRV-1 (Table S3 and Fig. S1A). By sequencing the assembled viral genome, including the 5′ and 3′ genome termini, the complete genome of WWPSRV-1 was confirmed to be 12,332 nucleotides (nt) in length, excluding poly-A (Fig. 1A and 2A). G+C pairs comprised 39.94% of the nucleotides. The WWPSRV-1 genome contained four ORFs in 5′to 3′ orientation, located at nt positions 1,003 to 8,739, 8,711 to 9,385, 9,367 to 10,947, and 10,940 to 12,007, respectively (Table 2). The four deduced ORFs of WWPSRV-1 were not in the same frame. ORF1 and ORF3 were in the same frame, while ORF2 and ORF4 were in the other frame. ORF1 to 4 were overlapping by a fragment (range from 2 to 23 bp, excluding the start and stop codons), respectively (Table 2 and Fig. 2A). The leader and trailer regions of the WWPSRV-1 genome were 1,002 and 325 nt in length, respectively (Table 2). A palindromic structure existed in the 5′ leader regions (Fig. 2A). The trailer regions ended with poly-A. Transcriptome sequencing (RNA-seq) reads of the three wasps mapped to the WWPSRV-1 genome exhibited similar fluctuating distribution on the viral genomic RNA, with a lower coverage close to 5′ ends of the genome (Fig. 2A).

**TABLE 2:** Features of the ORFs encoded by (+)ssRNA viruses discovered in the transcriptomes of weevil wasps^*a*^

Virus	ORF	ORF genomic location (nt)	Length (nt | aa)	Protein mass (kDa)	Isoelectric point	Signal peptide	Top BLASTX match	E value	Identity	Accession number
WWPSRV-1	ORF 1	1,003 to 8,739	7,737 | 2,578	295.59	8.05	−	RNA-dependent RNA polymerase (Nasonia vitripennis virus)	3E−143	78.15%	ACN94444.1
	ORF 2	8,711 to 9,385	675 | 224	25.73	4.79	−	ORF3 (Nasonia vitripennis virus)	3E−44	47.59%	ACN94446.1
	ORF 3	9,367 to 10,947	1,581 | 526	58.01	9.04	−	ORF4 (Nasonia vitripennis virus)	0	78.60%	ACN94448.1
	ORF 4	10,940 to 12,007	1,068 | 355	38.20	4.48	−	ORF5 (Nasonia vitripennis virus)	0	75.64%	ACN94449.1
WWPSRV-2	ORF	34 to 9,228	9,195 | 3,064	347.01	6.19	−	Polyprotein (Diabrotica virgifera virgifera virus 1)	0	40.81%	APF29088.1
AcPSRV−1	ORF 1	183 to 4,343	4,161 | 1,386	159.61	8.04	−	Nonstructural polyprotein (*Dicistroviridae* sp.)	2E−162	36.21%	QJI52218.1
	ORF 2	4,660 to 7,302	2,643 | 880	99.19	6.59	−	Structural polyprotein (black queen cell virus)	7E−136	36.23%	ASS83231.1

a −, does not exist.

10.1128/mSphere.00331-21.1 FIG S1 Assemble strategy view of the contigs from the different wasps’ transcriptomes. Download FIG S1, TIF file, 0.4 MB. Copyright © 2021 Wang et al. 2021 Wang et al. https://creativecommons.org/licenses/by/4.0/ This content is distributed under the terms of the Creative Commons Attribution 4.0 International license.

10.1128/mSphere.00331-21.10 TEXT S1 Contigs from these three wasps. AC, *Anisopteromalus calandrae*; LD, *Lariophagus distinguendus*; and TE, *Theocolax elegans*. Download Text S1, TXT file, 0.09 MB. Copyright © 2021 Wang et al. 2021 Wang et al. https://creativecommons.org/licenses/by/4.0/ This content is distributed under the terms of the Creative Commons Attribution 4.0 International license.

10.1128/mSphere.00331-21.7 TABLE S1 Primers used in this study. Download Table S1, XLSX file, 0.02 MB. Copyright © 2021 Wang et al. 2021 Wang et al. https://creativecommons.org/licenses/by/4.0/ This content is distributed under the terms of the Creative Commons Attribution 4.0 International license.

**(ii) WWPSRV-2.** Four contigs (AC_Contig_3, LD_Contig_2, and TE_Contig_2 and 3) (Text S1) were assembled into a big virus-like contig (4,899 bp), which had a high similarity with Diabrotica virgifera virgifera virus 1 (DvvV-1) polyprotein (coverage range from amino acid [aa] 1,440 to aa 2,995) (Table S3 and Fig. S1B). Moreover, AC_Contig_2 (3,308 bp) (Text S1) was also aligned to DvvV-1 polyprotein (coverage range from aa 210 to aa 1,002) (Table S3). By sequencing these contigs, the five contigs were verified to belong to the same virus, named WWPSRV-2 (Fig. 1B). The complete genome of WWPSRV-2 was 9,409 nt in length, excluding poly-A. G+C pairs comprised 40.95% of the nucleotides. Only one large ORF was predicted in WWPSRV-2 genome, located at nt 34 to 9,228 in 5′ to 3′ direction. The 5′ and 3′ untranslated regions (UTR) of WWPSRV-2 genome were 33 and 181 nt in length, respectively (Fig. 2A and Table 2). The fluctuating distributions of the three read maps were similar, with the higher coverage area near 3′ ends of the viral genome. However, a small number RNA-seq reads of *L. distinguendus* could map to the WWPSRV-2 genome (Fig. 1A). Of note, PCR detection results revealed that this virus could exist in the adults of *S. oryzae* (Fig. 1B and Fig. S2).

10.1128/mSphere.00331-21.2 FIG S2 PCR detection of the six viruses in the *Sitophilus oryzae* adults. In lanes 1 to 7 were the detection results from using specific primers from WWPSRV-1, WWPSRV-2, AcPSRV-1, AcNSRV-1, AcNSRV-2, LdNSRV-1, and *actin* (weevil), respectively. The primers used to detect viral genome sequence are shown in Table S1. Download FIG S2, TIF file, 0.3 MB. Copyright © 2021 Wang et al. 2021 Wang et al. https://creativecommons.org/licenses/by/4.0/ This content is distributed under the terms of the Creative Commons Attribution 4.0 International license.

**(iii) AcPSRV-1.** AC_Contig_4 (Text S1) was 7,558 bp in length, including poly-A, and contained regions similar with viral RNA-dependent RNA polymerase (RdRp) of *Dicistroviridae* sp. (Table S3). It contained two large ORFs in the 5′ to 3′ direction, located at nt positions 183 to 4,343 and 4,660 to 7,302, respectively. The contig was provisionally named AcPSRV-1, and G+C pairs comprised 34.10% of the nucleotides. The 5′UTR, intergenic region (IGR), and 3′UTR of AcPSRV-1 genome were 182, 242, and 247 nt in length, respectively (Fig. 2A and Table 2). However, this contig failed to be confirmed by PCR. Only the RNA-seq reads of *A. calandrae* could map to AcPSRV-1 genome. The higher coverage area also appeared close to 3′ ends of the viral genome (Fig. 2A).

### AcNSRV-1, AcNSRV-2, and LdNSRV-1 were related to negative-strand RNA virus.

**(i) AcNSRV-1.** Four contigs related to negative-strand RNA virus were discovered in *A. calandrae*. The two long contigs (AC_Contig_5, 12,699 bp; AC_Contig_6, 12,118 bp) (Text S1), which both had high identities with Linepithema humile rhabdo-like virus 1, were provisionally designated AcNSRV-1 and AcNSRV-2, respectively (Table S3). By sequencing, the confirmed complete genome of AcNSRV-1 was determined to be 12,702 nucleotides in length (Fig. 1C and 2B). G+C pairs comprised 37.85% of the nucleotides. The genome contained five large ORFs in the 3′ to 5′ direction, located at nt positions 209 to 1,498, 1,584 to 2,957, 3,081 to 3,479, 3,920 to 5,536, and 6,089 to 12,265, respectively (Table 3 and Fig. 2B). The fluctuating distribution of RNA-seq reads of *A. calandrae* exhibited that reads in the five ORF regions were much more numerous than those in the nearby untranslated region. The position of the peak corresponded exactly to the position of each ORF (Fig. 2B). The leader and trailer regions of the AcNSRV-1 genome were 208 and 437 nt in length, respectively. Their terminal nucleotides were complementary (Table 3 and Fig. 2B) and could form a putative panhandle structure which may be involved in genome replication (17).

**TABLE 3:** Features of the ORFs encoded by (−)ssRNA viruses discovered in the transcriptomes of weevil wasps^*a*^

Virus	ORF	Protein	ORF genomic location (nt)	Length (nt | aa)	Protein mass (kDa)	Isoelectric point	Signal peptide	No. of glycosylation sites		No. of phosphorylation sites			Top BLASTX match	E value	Identity	Accession number
								O-linked	N-linked	Ser	Thr	Tyr				
AcNSRV-1	1	N	209 to 1,498	1,290 | 429	48.27	5.75	−	8	0	20	10	5	Putative capsid (Linepithema humile rhabdo-like virus 1)	6E−40	27.29%	AXA52563.1
	2	P	1,584 to 2,957	1,374 | 457	50.98	5.90	−	20	0	24	19	4	ND			
	3	M	3,081 to 3,479	399 | 132	14.87	6.89	−	1	0	8	4	3	ND			
	4	G	3,920 to 5,536	1,617 | 538	60.24	6.71	+	1	2	32	14	6	Putative glycoprotein (Hubei rhabdo-like virus 3)	2E−19	25.50%	YP_009336887.1
	5	L	6,089 to 12,265	6,177 | 2,058	236.10	7.78	−	16	0	104	67	32	Putative RdRp-complex (Linepithema humile rhabdo-like virus 1)	0	40.27%	AXA52562.1
AcNSRV-2	1	N	29 to 1,330	1,302 | 433	47.77	6.43	−	0	0	13	11	6	Putative capsid (Linepithema humile rhabdo-like virus 1)	7.00E−53	30.75%	AXA52563.1
	2	P	1,518 to 2,624	1,107 | 368	40.35	5.32	−	21	0	30	17	5	ND			
	3	M	2,803 to 3,183	381 | 126	14.23	7.75	−	0	0	6	9	5	ND			
	4	G	3,453 to 5,075	1,623 | 540	60.08	8.36	+	7	5	33	19	9	Glycoprotein (Blattodean arli-related virus OKIAV101)	3.00E−25	25.12%	QMP82174.1
	5	P6	5,214 to 5,429	216 | 71	8.23	7.76	−	0	0	2	1	0	ND			
	6	L	5,572 to 11,886	6,315 | 2,104	239.37	6.84	−	14	0	91	65	38	Putative RdRp-complex (Linepithema humile rhabdo-like virus 1)	0	40.19%	AXA52562.1
LdNSRV-1	1	N	44 to 1,408	1,365 | 454	49.41	6.13	−	5	0	18	20	4	Nucleocapsid protein (hymenopteran rhabdo-related virus OKIAV109)	3.00E−99	39.81%	QMP82140.1
	2	P	1,499 to 2,587	1,089 | 362	39.51	6.98	−	21	0	25	12	3	ND			
	3	M	2,611 to 3,342	732 | 243	27.11	8.94	−	3	0	13	6	2	Hypothetical protein (hymenopteran rhabdo-related virus OKIAV109)	2.00E−18	33.51%	QMP82142.1
	4	G	3,542 to 5,080	1,539 | 512	57.70	7.66	+	2	2	32	21	2	Glycoprotein (hymenopteran rhabdo-related virus OKIAV109)	3.00E−48	28.07%	QMP82143.1
	5	L	5,169 to 11,519	6,351 | 2,116	238.53	8.42	−	28	0	133	61	23	RdRp (hymenopteran rhabdo-related virus OKIAV109)	0	48.73%	QMP82144.1

a +, exists; −, does not exist; ND, no significant homology was detected.

**(ii) AcNSRV-2.** The complete genome of AcNSRV-2 failed to be confirmed by PCR. The known length was 12,118 nucleotides and G+C pairs comprised 37.85% of the nucleotides. The genome contained six ORFs in the 3′ to 5′ direction, located at nt positions 29 to 1,330, 1,518 to 2,624, 2,803 to 3,183, 3,453 to 5,075, 5,214 to 5,429, and 5,572 to 11,886, respectively (Table 3 and Fig. 2B). The six ORFs were also confirmed by the RNA-seq reads fluctuating distribution (Fig. 2B). The leader and trailer regions of the AcNSRV-2 genome were 28 and 232 nt in length, respectively. Their terminal nucleotides were not complementary.

**(iii) LdNSRV-1.** LD_Contig_3 (10,832 bp) in *L. distinguendus*, designated LdNSRV-1, showed a certain sequence similarity with a rhabdovirus, Wuhan ant virus. The complete genome of LdNSRV-1 verified by sequencing was 11,633 nucleotides in length with G+C pairs comprising 45.64% of the nucleotides (Fig. 1D and 2B). It contained five large ORFs in the 3′ to 5′ direction, located at nt positions 44 to 1,408, 1,499 to 2,587, 2,611 to 3,342, 3,542 to 5,080, and 5,169 to 11,519, respectively (Table 3 and Fig. 2B). The fluctuating distribution of RNA-seq reads of *L. distinguendus* also indicated the existence of the five ORFs (Fig. 2B). The leader and trailer regions of the LdNSRV-1 genome were 43 and 114 nt in length, respectively. Their terminal nucleotides were not complementary.

In *T. elegans*, the contigs related to negative-strand RNA virus were too small to get the complete virus genome (Table S3).

### WWPSRV-1 represented a novel virus related to Nora virus.

General properties of all ORFs in WWPSRV-1 genome were listed in Table 2. The predicted translation products of WWPSRV-1 ORF1 to 4 were found to have amino acid sequences similar to those of the Nasonia vitripennis virus RdRp and ORF3, 4, and 5, respectively. ORF1 protein was predicted to be a large transmembrane polyprotein (2,578 aa and isoelectric point [pI] of 8.05) with three noncytoplasmic domains, two cytoplasmic domains, and four transmembrane regions (Fig. S3A). A specified putative helicase domain (aa 824 to 1,005), a trypsin-like serine protease domain (aa 1,773 to 1,995), and a RdRp domain (aa 2,157 to 2,530) were successively existent in the noncytoplasmic domain of ORF1 protein (Fig. 2A and Fig. S4).

10.1128/mSphere.00331-21.3 FIG S3 Graphical representation of the polyproteins encoded by the (+)ssRNA viruses identified in the wasps’ transcriptomes. (A) The polyprotein encoded by WWPSRV-1 ORF1. (B) The polyprotein encoded by WWPSRV-2 ORF. (C) The polyprotein encoded by AcPSRV-1 ORF1. Numbers represent the amino acid positions from the N terminus to C terminus. Download FIG S3, TIF file, 0.2 MB. Copyright © 2021 Wang et al. 2021 Wang et al. https://creativecommons.org/licenses/by/4.0/ This content is distributed under the terms of the Creative Commons Attribution 4.0 International license.

10.1128/mSphere.00331-21.4 FIG S4 Structure of helicase, protease, and RdRp domains of WWPSRV-1, WWPSRV-2, and AcPSRV-1. (A) Partial amino acids containing helicase motifs A to C are shown with their secondary structures (red cylinders, α-helices; green arrows, β-strands). Sequence logos for helicase motifs A to C of 12 positive-strand RNA viruses belonging to the families *Dicistroviridae* (ABPV, CrPV, TrV, and BQCV), *Iflaviridae* (DWV, SBV, SBPV, and ApIV), and unclassified Nora virus-related viruses (HaNV, AiNV, ANV, and DiNV) are shown with the corresponding sequences of WWPSRV-1, WWPSRV-2, and AcPSRV-1. (B) The three-dimensional structural models of the helicase domains of these three viruses predicted by the Phyre2 web. The positions of motifs A to C are marked. Motif A, magenta; motif B, red; motif C, blue. Confidence in the models of WWPSRV-1, WWPSRV-2, and AcPSRV-1: 172 residues (95%) modeled at >90% accuracy, 166 residues (97%) modeled at >90% accuracy, and 178 residues (98%) modeled at >90% accuracy, respectively. (C) Multiple sequence alignment of the putative protease domains of these 3 viruses and 12 other picorna-like viruses. Identical residues are shaded in dark blue and similar residues are in light blue. (D) Partial amino acid containing RdRp motifs A to H are shown with their secondary structures (red cylinders, α-helices; green arrows, β-strands). Sequence logos for RdRp motifs A to H of 12 positive-strand RNA viruses are shown with the corresponding sequences of these 3 viruses. (E) The three-dimensional structural models of the RdRp domains of these three viruses predicted by the Phyre2 web. The positions of motifs A to H are marked. Motif A, magenta; motif B, orange; motif C, yellow; motif D, cyan; motif E, wheat; motif F, blue; motif G, red; motif H, white. Confidence in the models of WWPSRV-1, WWPSRV-2, and AcPSRV-1: 374 residues (100%) modeled at >90% accuracy, 442 residues (100%) modeled at >90% accuracy and 388 residues (100%) modeled at >90% accuracy, respectively. Full names and references of these viruses are shown in Table S2. Download FIG S4, TIF file, 1.4 MB. Copyright © 2021 Wang et al. 2021 Wang et al. https://creativecommons.org/licenses/by/4.0/ This content is distributed under the terms of the Creative Commons Attribution 4.0 International license.

ORF2 protein (244 aa and pI = 4.79) contained three coils, which was similar with tropomyosin, according to InterProScan. VIRALpro suggested that it was capsid protein (distance = 0.4141) and had the tail sequence (distance = 0.1172). ORF3 (526 aa and pI = 9.04) and ORF4 (355 aa and pI = 4.48) proteins were also suggested to encode capsid proteins (distance = 1.0846 and 0.8483, respectively).

**FIG 3:**
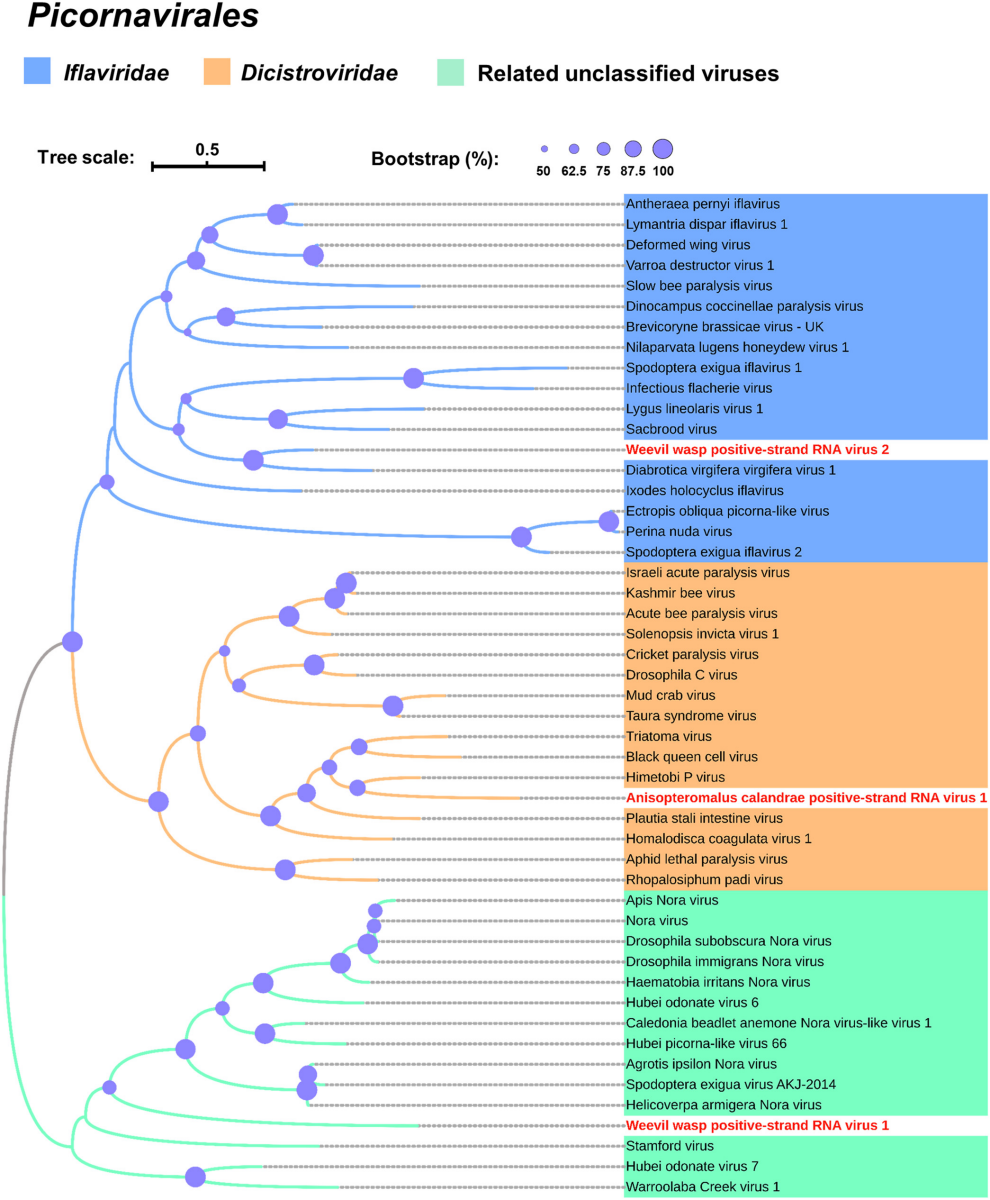
Phylogenetic analysis of positive-strand RNA viruses discovered in the parasitoid wasps’ transcriptomes. Maximum likelihood phylogenetic tree was constructed based on the deduced RdRp domain. Viruses are from the order *Picornavirales* and the GenBank accession numbers are shown in Table S2. Purple circles on the branches of each tree relate to 50 to 100% bootstrap support calculated from 1,000 replicates.

To determine the relationship of WWPSRV-1 with other picorna-like viruses, we conducted a maximum likelihood phylogenetic analysis based on the amino acid core sequences of RdRp. Notably, WWPSRV-1 clustered as a distinct lineage in Nora virus-related clade, indicating the need for a novel virus genus or family (Fig. 3).

10.1128/mSphere.00331-21.8 TABLE S2 Viral sequences used for phylogenetic analysis, sequence alignments, and protein prediction in this study. Download Table S2, XLSX file, 0.01 MB. Copyright © 2021 Wang et al. 2021 Wang et al. https://creativecommons.org/licenses/by/4.0/ This content is distributed under the terms of the Creative Commons Attribution 4.0 International license.

### WWPSRV-2 was a novel iflavirus.

Only a big ORF was predicted in WWPSRV-2 genome, which encoded a large polyprotein. The putative domains in the polyprotein were arranged successively in the order CP1, CP2, CP3, helicase, protease, and RdRp, corresponding to that in iflaviruses (Fig. 2A).

The polyprotein (3,064 aa and pI = 6.19) was a large transmembrane polyprotein that consisted of four noncytoplasmic domains, three cytoplasmic domains, and six transmembrane regions. All three predicted picornavirus-like capsid protein domains appeared in the first noncytoplasmic domain. A typical helicase domain (aa 1,440 to 1,611) was present in the third noncytoplasmic domain. Moreover, a relatively small motif (aa 1,799 to 1,842) predicted as a zinc finger domain was also in that noncytoplasmic domain. The putative protease (aa 2,212 to 2,417) and RdRp (aa 2,490 to 2,931) domains were in the last noncytoplasmic domain (Table 2 and Fig. S3B and S4).

To determine the phylogenetic relationship of WWPSRV-2 in other iflaviruses, phylogenetic analysis was performed using the highly conserved RdRp region. WWPSRV-2 could form a clade with the known members of iflaviruses, such as sacbrood virus (SBV) and Lygus lineolaris virus 1 (LyLV-1) (Fig. 3).

### AcPSRV-1 was a novel dicistrovirus.

Two big ORFs were present in the AcPSRV-1 genome, which encoded nonstructural and structural proteins, corresponding to that in dicistroviruses. The nonstructural protein encoded by ORF1 was a large transmembrane polyprotein (1,386 aa and pI = 8.04) that consisted of two noncytoplasmic domains, one cytoplasmic domain, and two transmembrane regions. The helicase (aa 67 to 248), protease (aa 595 to 831), and RdRp (aa 963 to 1,350) domains were in the different two noncytoplasmic domains (Fig. S3C and S4). The structural protein (880 aa and pI = 6.59) encoded by ORF2 was a polyprotein in which three picornavirus-like capsid protein domains exist (Fig. 2A and Table 2).

Phylogenetic analysis of the highly conserved RdRp region was performed to confirm the relationship of AcPSRV-1 in other dicistroviruses. AcPSRV-1 could be clustered with Himetobi P virus (HiPV), black queen cell virus (BQCV), and Triatoma virus (TrV), which belong to genus *Triatovirus* (Fig. 3).

### Gene junction analysis of the three negative-strand RNA viruses.

By comparing the 5′ and 3′ untranslated regions and intergenic regions of the AcNSRV-1, AcNSRV-2, and LdNSRV-1, we pinpointed for each ORF the putative termination signals (TS), intergenic spacer (IS), and transcription initiation (TI). A highly conserved motif was identified in the 5′ and 3′ untranslated regions and intergenic regions of the AcNSRV-1, AcNSRV-2, or LdNSRV-1, respectively.

**FIG 4:**
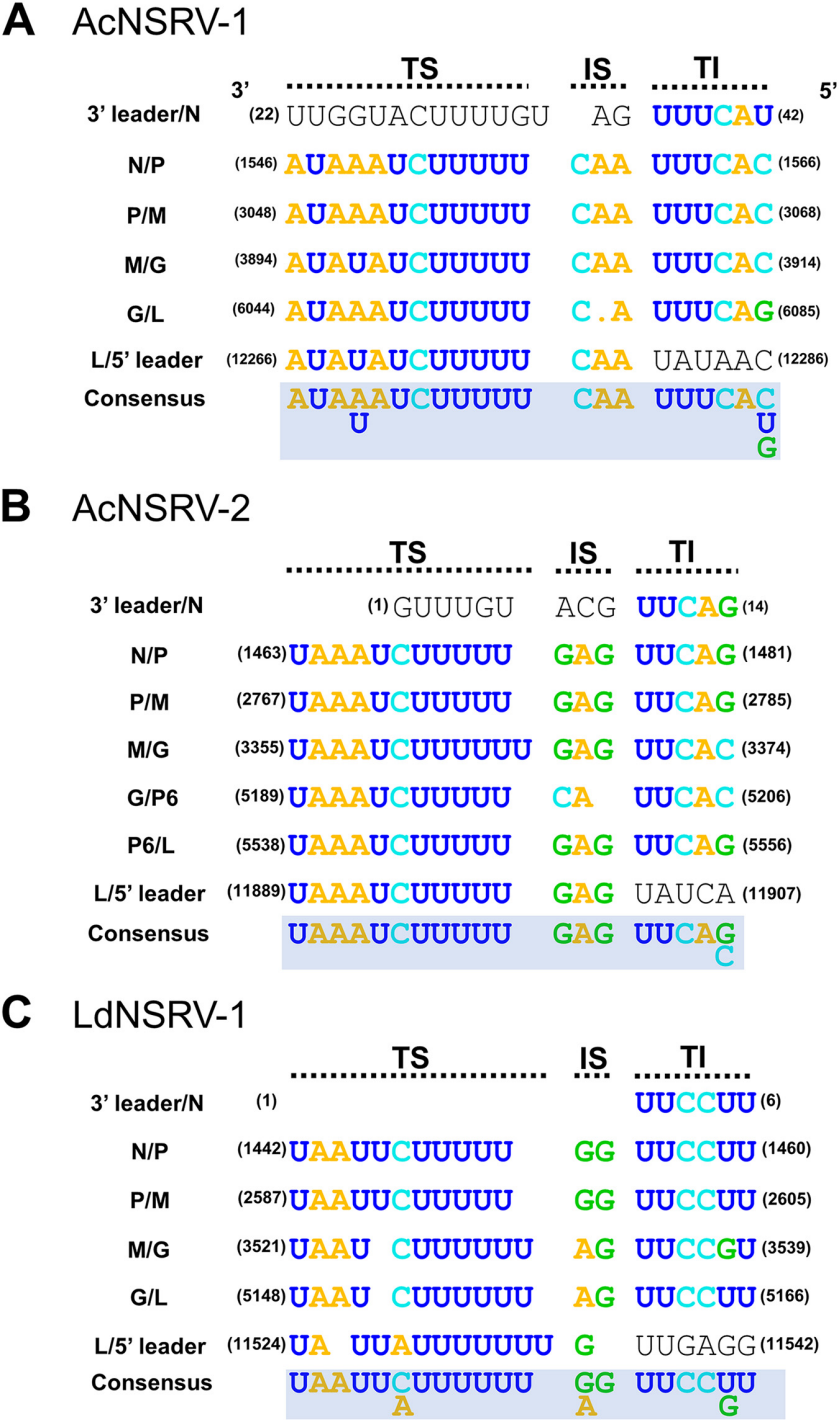
Comparison of putative gene junction regions between ORFs in AcNSRV-1 (A), AcNSRV-2 (B), and LdNSRV-1 (C). Alignments of the putative gene junction sequences are shown in a 3′ to 5′ orientation in the negative strand. TS, termination signals; IS, intergenic sequence; TI, transcription initiation. The consensus motif is shown below the sequences for each viral ORF. Numbers at the beginning of the sequences represent the nucleotide positions from in a 3′ to 5′ orientation.

In AcNSRV-1 genome, the conserved transcription initiation motif of 3′-UUUCA(C/U/G)-5′ was identified upstream of each putative ORF. The transcription termination motif of 3′-AUA(A/U)AUCUUUUU-5′ was detected downstream of every ORF. The conserved intergenic spacer 3′-C(.)A-5′ was found in each intergenic region (Fig. 4A).

In AcNSRV-2 genome, the conserved transcription initiation motif of 3′-UUCA(G/C)-5′ was identified upstream of each putative ORF, and the transcription termination motif of 3′-UAAAUCUUUUU(U)-5′ was detected downstream of every ORF. The conserved intergenic spacer 3′-GAG-5′ was found in the intergenic region among N/P, P/M, and M/L (Fig. 4B).

In LdNSRV-1 genome, each ORF was separated by a highly conserved gene junction with the consensus 3′-UAAU(U)CUUUUU(U)(G/A)GUUCC(U/G)U-5′. The conserved transcription initiation motif of 3′-UUCC(U/G)U-5′ was identified upstream of each putative ORF. The transcription termination motif of 3′-UA(A)U(U)(C/A)UUUUU(U)(U)-5′ was detected downstream of every ORF. The conserved intergenic spacer 3′-(G/A)(G)-5′ was found in each intergenic region (Fig. 4C).

### AcNSRV-1 and AcNSRV-2 were novel viruses belonging to family *Lispiviridae*.

The general properties of the tentative proteins encoded by the AcNSRV-1 and AcNSRV-2 genome were shown in Table 3. Both the ORF1 proteins encoded by the two viruses had close to 30% identities with the putative capsid of Linepithema humile rhabdo-like virus 1. Through the VIRALpro analysis, the ORF1 proteins were suggested to have the capsid sequences (distance was 0.5556 in AcNSRV-1 and distance was 1.2285 in AcNSRV-2). Accordingly, the ORF1 proteins of the two viruses were supposed to be the putative nucleocapsid proteins (N), which had similarity in length (1,290 aa and 1,302 aa, respectively) and isoelectric point (5.75 and 6.43, respectively).

Only ORF4 proteins encoded by the two viruses were predicted to be transmembrane polyproteins and to possess a cleavable signal peptide. Compared to the three transmembrane regions present in the AcNSRV-1 ORF4 protein, only one was in the AcNSRV-2 ORF4 protein (Fig. S5A and B). The AcNSRV-1 ORF4 protein had 25.5% identity with the putative glycoprotein of Hubei rhabdo-like virus 3. In addition, it was classified to the fusion glycoprotein F0, *Paramyxoviridae* (E value = 1.00E−08), within the InterProScan. The AcNSRV-2 ORF4 protein showed 25.12% identity with the putative glycoprotein of Blattodean arli-related virus OKIAV101. Moreover, only the ORF4 proteins of the two viruses were recommended to have the potential N-linked sites. All of these results indicated that ORF4 encoded the viral glycoprotein (G).

10.1128/mSphere.00331-21.5 FIG S5 Graphical representation of the G proteins encoded by the (−)ssRNA viruses identified in the wasps’ transcriptomes. (A) The polyprotein encoded by AcNSRV-1 ORF4. (B) The polyprotein encoded by AcNSRV-1 ORF4. (C) The polyprotein encoded by LdNSRV-1 ORF4. Numbers represent the amino acid positions from the N terminus to C terminus. Download FIG S5, TIF file, 0.2 MB. Copyright © 2021 Wang et al. 2021 Wang et al. https://creativecommons.org/licenses/by/4.0/ This content is distributed under the terms of the Creative Commons Attribution 4.0 International license.

The AcNSRV-1 ORF5 and the AcNSRV-1 ORF6 were supposed to encode the viral large polymerase proteins (L). Both L proteins exhibited more than 40% similarity with the putative RdRp-complex of Linepithema humile rhabdo-like virus 1. Through the InterProScan, the putative RdRp, mRNA-capping enzyme (Cap, or named polyribonucleotidyltransferase, PRNTase), and methyltransferase (MT) domains were successively arranged in the L protein (Fig. 2B and Fig. S6).

10.1128/mSphere.00331-21.6 FIG S6 Structure of the RdRp, PRNTase, and methyltransferase domains of AcNSRV-1, AcNSRV-2, and LdNSRV-1. (A) Partial amino acid containing RdRp motifs A to F and GHP (Gly-His-Pro) are shown with their secondary structures (red cylinders, α-helices; green arrows, β-strands). Sequence logos for RdRp motifs A to G and GHP of 12 negative-strand RNA viruses belonging to the families *Lispiviridae* (HbRLV-3, TcTV-6, LHRLV-1, BerV, IARV-OKIAV103, and BARV-OKIAV102) and *Rhabdoviridae* (WHFV-2, SyFV-3, LNV-2, HyRRV-OKIAV38, SFRV, and LeRRV-OKIAV34) are shown with the corresponding sequences of AcNSRV-1, AcNSRV-2, and LdNSRV-1. (B) The three-dimensional structural models of the RdRp domains of these three viruses predicted by the Phyre2 web. The positions of motifs A to F and GHP are marked. Motif GHP, red; motif A, yellow; motif B, magenta; motif C, cyan; motif D, orange; motif E, white; motif F, blue. Confidence in the models of AcNSRV-1, AcNSRV-2, and LdNSRV-1: 828 residues (100%) modeled at >90% accuracy, 851 residues (99%) modeled at >90% accuracy, and 877 residues (100%) modeled at >90% accuracy, respectively. (C) Partial amino acids containing PRNTase motifs A to E are shown with their secondary structures (red cylinders, α-helices; green arrows, β-strands). Sequence logos for PRNTase motifs A to E of 12 negative-strand RNA viruses are shown with the corresponding sequences of these three viruses. (D) The three-dimensional structural models of PRNTase domains of these three viruses predicted by the Phyre2 web. The positions of motifs A to E are marked with orange. Confidence in the models of AcNSRV-1, AcNSRV-2, and LdNSRV-1: 235 residues (100%) modeled at >90% accuracy, 235 residues (100%) modeled at >90% accuracy, and 237 residues (100%) modeled at >90% accuracy, respectively. (E) Partial amino acid containing methyltransferase motifs (I to IV and VI to X) are shown with their secondary structures (red cylinders, α-helices; green arrows, β-strands). Sequence logos for methyltransferase motifs of 12 negative-strand RNA viruses are shown with the corresponding sequences of these three viruses. (F) The three-dimensional structural models of the methyltransferase domains of these three viruses predicted by the Phyre2 web. KDKE observed in motif X, motif IV, motif VI, and motif VIII, are marked with red, magenta, cyan, and orange, respectively. Motif I is marked with blue. Confidence in the models of AcNSRV-1, AcNSRV-2, and LdNSRV-1: 195 residues (100%) modeled at >90% accuracy, 197 residues (100%) modeled at >90% accuracy, and 194 residues (100%) modeled at >90% accuracy, respectively. Full names and references of these viruses are shown in Table S2. Download FIG S6, TIF file, 2.2 MB. Copyright © 2021 Wang et al. 2021 Wang et al. https://creativecommons.org/licenses/by/4.0/ This content is distributed under the terms of the Creative Commons Attribution 4.0 International license.

Based on the conserved genomic orientation of mononegaviruses (3′-N-P-M-G-L-5′), ORF2 protein of AcNSRV-1 (pI = 5.90) or AcNSRV-2 (pI = 5.32), containing 47 or 52 potential phosphorylation sites, was most likely the phosphoprotein (P). ORF3 protein of AcNSRV-1 (pI = 6.89) or AcNSRV-2 (pI = 7.75), containing 15 or 20 phosphorylation sites, may be a phosphorylated matrix protein (M). In addition, the AcNSRV-2 had an additional small protein P6 (71 aa and pI = 7.76) lying between the G and L proteins. This was also confirmed by the RNA-seq reads’ fluctuating distribution (Fig. 2B).

**FIG 5:**
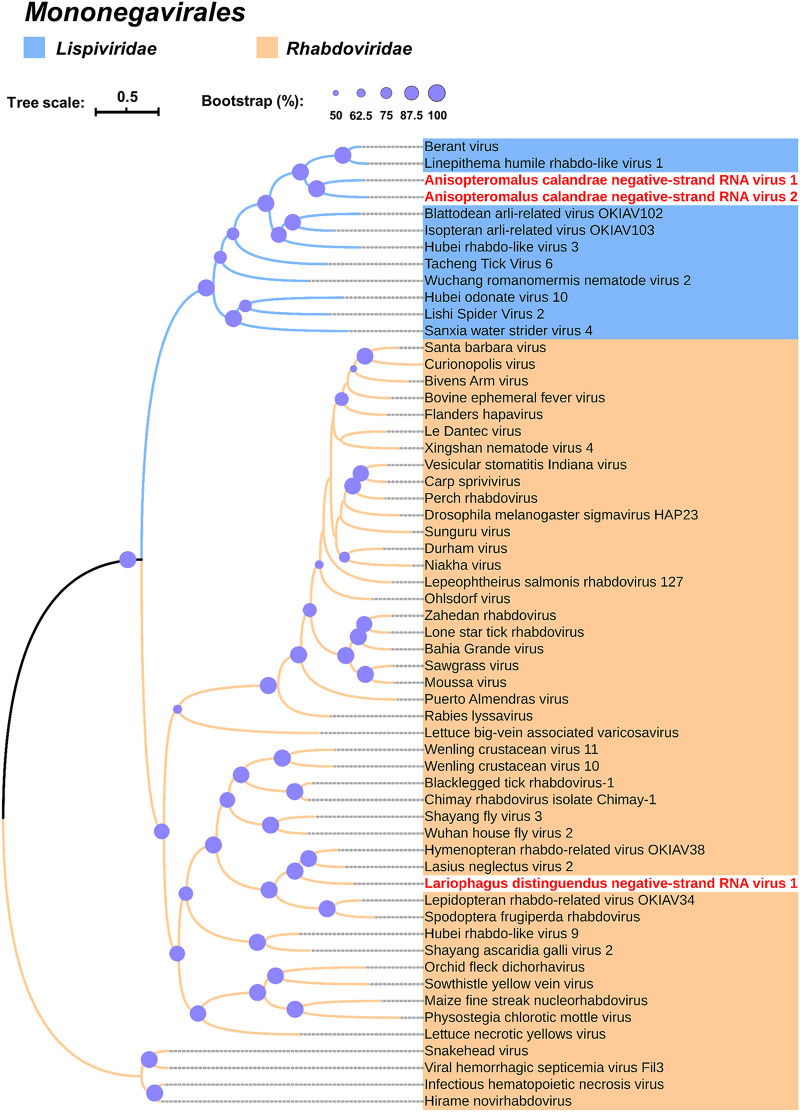
Phylogenetic analysis of negative-strand RNA viruses discovered in the parasitoid wasps’ transcriptomes. Maximum likelihood phylogenetic tree was constructed based on core motif in L protein. Viruses are from the order *Mononegavirales* and the GenBank accession numbers are shown in Table S2. Purple circles on the branches of each tree relate to 50 to 100% bootstrap support calculated from 1,000 replicates.

Phylogenetic analysis of the highly conserved region of L protein was performed to confirm the relationship of AcNSRV-1 and AcNSRV-2 in other lispiviruses. AcNSRV-1 and AcNSRV-2 could be clustered with Berant virus and Linepithema humile rhabdo-like virus 1, which belong to the family *Lispiviridae* (Fig. 5).

### LdNSRV-1 was a novel rhabdovirus.

The general properties of the tentative proteins encoded by the LdNSRV-1 genome were shown in Table 3. The LdNSRV-1 ORF1 protein had a close-to-39.81% identity with nucleocapsid protein of hymenopteran rhabdo-related virus OKIAV109 (HyRRV-OKIAV109). It was suggested to have the capsid sequence (distance = 0.3497) with VIRALpro. Accordingly, the ORF1 protein was supposed to be the N protein. ORF4 protein was predicted to be a transmembrane polyprotein which only contained one transmembrane region and to possess a cleavable signal peptide (Fig. S5C). It has 28.07% identity with HyRRV-OKIAV109 glycoprotein. Moreover, ORF4 protein was recommended to have two potential N-linked sites. All of these indicated that ORF4 encoded the viral G protein. The LdNSRV-1 ORF5 was supposed to encode the viral L protein which also contained the putative RdRp, PRNTase, and MT domains (Fig. 2B and Fig. S6). It exhibited more than 40% similarity with HyRRV-OKIAV109 RdRp-complex. The LdNSRV-1 ORF2 protein (pI = 6.98), containing 40 putative phosphorylation sites, was most likely a P protein. ORF3 protein (pI = 8.94), containing 21 putative phosphorylation sites, may be an M protein.

Phylogenetic analysis of the highly conserved region of L protein was performed to confirm the relationship of LdNSRV-1 in other rhabdoviruses. LdNSRV-1 could be clustered with Lasius neglectus virus 2 and hymenopteran rhabdo-related virus OKIAV38, which belong to the genus *Alphahymrhavirus* (Fig. 5).

## DISCUSSION

More and more RNA viruses have been discovered with the increasing transcriptome data of insects. Also, whole-transcriptome analysis has become a very important method in virus identification and surveillance in insects relevant to economics and public health. For example, 42 putative novel picornaviruses are discovered in varroa-free Australian Apis mellifera (18), three novel RNA viruses are identified in *Aedes vexans nipponii* collected at a densely populated district of Seoul, and many insect-specific viruses, belonging to *Baculoviridae*, *Nimaviridae*, and *Iflaviridae*, are found in Culex quinquefasciatus and Culex tritaeniorhynchus gathered in China and Kenya (11, 19). In this study, we identified six novel RNA viruses from three species of hymenopteran Pteromalidae parasitoids of the rice weevil *S. oryzae.* We characterized organization of viral genomes, their phylogenetic locations, and structures of viral nonstructural protein. These results indicate that the positive-strand and negative-strand RNA viruses could be simultaneously present in these three wasps.

Picornaviruses identified here are WWPSRV-1, WWPSRV-2, and AcPSRV-1. The organization of these viral genomes varies greatly. WWPSRV-1 possesses four ORFs in the genome, whereas WWPSRV-2 and AcPSRV-1 possess monocistronic and dicistronic genomes, respectively. In addition, these three viruses have the similar fluctuating distribution of sequencing reads, with the higher coverage area near 3′ ends of the viral genome (Fig. 2A). The 3′ bias of the viral genome coverage is likely to be attributed to the expression of subgenomic RNAs (sgRNAs) (20). However, it also cannot be ignored that RNA degradation or shearing during sample handling and RNA extraction may also lead to a controllable 3′ bias in short-read RNA-seq data (21). The analysis of PCR results and transcriptome data (Fig. 1A and B and Fig. 2A) confirms that WWPSRV-1 and WWPSRV-2 can be detected in these three wasps. The observations demonstrate that these two viruses may have the possibility of interspecies virus transmission. Just like Drosophila C virus (DCV), they can infect a much broader range of *Drosophila* species through horizontal transmission (22). Significantly, WWPSRV-2, which belong to family *Iflaviridae* of which some viruses can result in lethal infections in silkworms (23) and honeybees (24), can also be detected in the wasp host rice weevil. Moreover, it has been reported that an iflavirus Dinocampus coccinellae paralysis virus, found in Dinocampus coccinellae wasps which parasitize the lady beetle Coleomegilla maculata, can replicate in lady beetle cerebral ganglia and thus induce changes in lady beetle behavior such as tremors, gait disturbance, and limitations in movement (25). Accordingly, WWPSRV-2 may also play a role in the virus-wasp-weevil interaction. WWPSRV-1 shows high sequence similarity with Nasonia vitripennis virus, for which the incomplete genome was identified from cDNA libraries of parasitoid wasps Nasonia vitripennis (26). In the phylogenetic tree, WWPSRV-1 could cluster as a distinct lineage in Nora virus-related clade. In this clade, the most studied virus is Nora virus, which is a persistent nonpathogenic virus that infects various *Drosophila* species (27, 28). However, Nora virus-infected D. melanogaster exhibits an increase in immune-related gene expression over time (29). AcPSRV-1 has a dicistronic genome with gene organization similar to that of dicistroviruses. Phylogenetic analyses of the RdRp domain revealed that, clustering with Himetobi P virus (detected in the small brown planthopper Laodelphax striatellus) as closest relative, AcPSRV-1 belongs to genus *Triatovirus* (30). In this genus, black queen cell virus, which also infects honeybees (hymenopteran insect), causes broad health problems with risk of colony sustainability (31).

Mononegaviruses identified here are AcNSRV-1, AcNSRV-2, and LdNSRV-1. AcNSRV-1 and AcNSRV-2 belong to family *Lispiviridae* of the order *Mononegavirales*, whereas LdNSRV-1 belongs to a big family *Rhabdoviridae*. Sixteen viral genome sequences which belong to family *Lispiviridae* (3, found in hymenopteran orussidae, cynipidae, or aphelinidae insects, respectively) and *Rhabdoviridae* (13, found in hymenopteran eulophidae, sphecidae, chrysididae, pompilidae, sapygidae, or mymaridae insects, respectively) have been identified in the hymenopteran insects (32–34). The impact of these viruses on the host wasps has rarely been determined. However, some insects, such as flies, mosquitos, and aphids, when infected by the rhabdoviral sigmaviruses can become paralyzed or die after exposure to high concentrations of CO_2_, whereas uninfected hosts can recover (27, 35, 36).

The arrangement of the genomes of AcNSRV-1 and LdNSRV-1 follows the typical basic five-ORF pattern (3′-N-P-M-G-L-5′) of mononegaviral genomes (37). The mRNA of each gene can be synthesized directly from the viral genome (38). The high-coverage area of sequencing reads of AcNSRV-1 and LdNSRV-1 reveals that the N, P, M, and G may have a high expression level. Moreover, AcNSRV-2 contains an additional P6 protein between G and L proteins (Fig. 2B). The small additional proteins interposed between the typical basic protein were often identified in the plant rhabdoviruses (39). Some of these proteins’ functions have been made clear, e.g., P3 acts as a movement protein in rice transitory yellowing virus (RTYV) and rice yellow stunt rhabdovirus (RYSV) and P6 acts as a systemic RNA silencing suppressor in RYSV (40–42). In addition, a highly conserved motif in the intergenic junction which could be grouped into TS, IS, and TI is predicted to be present in all three viruses. Moreover, the 3′ and 5′ end sequences of AcNSRV-1 are complementary and can form a putative panhandle structure thought to be involved in genome replication (17).

In conclusion, we have expanded the diversity of RNA viruses in the parasitoid wasps, and these RNA viruses may play an important role in the biocontrol of rice weevils. For better application of this information, much work should be accomplished. Just like PpNSRV-1 (12), it also should be confirmed whether these viruses can affect the parasitism rate and the life span of parasitoid wasps. Moreover, the virus transmission among wasps and their host weevil should also be investigated.

## MATERIALS AND METHODS

### Insect rearing.

Rice weevils (*S. oryzae*) were initially collected from a cereal storehouse in Hangzhou, China in 2017. They were successively reared on wheat seeds in the laboratory at 30 ± 1°C, 80% relative humidity, and 12-h light/12-h dark photoperiod. The mated adult beetles were provided with the fresh wheat seeds to lay eggs for a week (the seeds were replaced weekly). After 10 to 15 days, eggs developed into elder larvae used as hosts for the wasps. The colonies of A. calandrae, L. distinguendus, and T. elegans were initially collected from the parasitized rice weevil hosts of the same sites. The adults of the three wasp species were independently reared on 10% (vol/vol) honey at 25 ± 1°C, 50% relative humidity, 12-h light/12-h dark photoperiod. For parasitism, the mated adults of the three wasp species were placed together with the wheat seeds that contained the host larvae. These parasitized beetle larvae were reared in the same conditions as were the wasp adults. The adults of *A. calandrae* or *L. distinguendus* emerged after 15 days. The adults of *T. elegans* emerged after 20 days. Each wasp colony was independently reared in the laboratory at least three generations before the experiments.

### Next-generation sequencing, sequence assembly, and virus-related sequence discovery.

Total RNA from 20 female adults of *A. calandrae*, *L. distinguendus*, or *T. elegans* was extracted using TRIzol (Invitrogen, CA, USA). The RNA quality was assessed using NanoDrop 2000 (ThermoFisher Scientific, Waltham, MA) and an Agilent 2100 bioanalyzer (ThermoFisher Scientific, CA, USA). RNA concentration was measured using an Invitrogen Qubit 2.0 Fluorometer (ThermoFisher Scientific, CA, USA). RNA samples (RNA integrity number > 8) were sent to Novogene company (Beijing, China) for the subsequent library construction. Sequencing libraries were generated using NEBNext Ultra RNA Library Prep kit for Illumina (NEB, USA) following the manufacturer’s recommendations and index codes were added to attribute sequences to each sample. The clustering of the index-coded samples was performed on a cBot Cluster Generation System using TruSeq PE cluster kit v3-cBot-HS (Illumia) according to the manufacturer’s instructions. After cluster generation, the library preparations were sequenced on an Illumina Hiseq platform and paired-end reads were generated. The low-quality, adaptor-polluted, and high content of unknown base (N) reads were removed. The clean reads were performed with *de novo* assembly using Trinity program (43). After assembly, the remaining contigs were annotated with BLASTX (E values ≤ 1e−5) in the NCBI nonredundant protein sequence databases to identify the virus-related sequences.

### Viral genome sequencing and detection.

Primers for viral genome sequencing and detection were designed based on the virus genome-like contigs (Table S1). Total RNA from adult female wasps or adult weevils was extracted using TRIzol. Single-stranded cDNA was synthesized from the RNA using the TransScript one-step gDNA removal and cDNA synthesis SuperMix kit (TransGen Biotech, Beijing, China). cDNA was used as a template for PCR. The terminal sequences of the viral genome were confirmed by 5′ or 3′ RACE according to the instructions of the SMART RACE cDNA amplification kit (Clontech, CA, USA). All amplified PCR products were cloned into pGEM-T Easy vectors (Promega, Beijing Branch, China) and sequenced.

### Bioinformatic and phylogenetic analysis.

Nucleotide sequence analysis and assembly were performed using DNAStar Lasergene software version 7.0 (Madison, WI, USA). Viral ORFs were predicted using open-source NCBI ORF finder (https://www.ncbi.nlm.nih.gov/orffinder/). Molecular weight and isoelectric point (pI) of the predicted ORFs were calculated via open-source ProtParam (https://web.expasy.org/protparam/). The viral proteins were queried through InterPro (http://www.ebi.ac.uk/interpro/) and Phyre2 for protein modeling, prediction, and analysis (44). ViralPro was used to determine whether the viral proteins were possibly capsid or tail proteins (45). Potential phosphorylation sites were determined by open-source NetPhos 3.1 (46). Glycosylation sites were determined by NetOGlyc 4.0 (47) and NetNGlyc 1.0 (https://services.healthtech.dtu.dk/service.php?NetNGlyc-1.0).

To identify putative conserved transcription termination and initiation sequences of (−)ssRNA viruses (negative-sense single-stranded RNA), noncoding viral genome regions were analyzed using the open-source MEME suite 5.3.0 for the motifs analysis (48).

To identify the viral genomic RNA distribution in these three wasps’ transcriptomes, clean RNA-seq reads from all three wasps were mapped to the complete genome of the six novel RNA viruses using the CLC Genomics Workbench 12.

Multiple alignments of amino acid sequences were performed using Clustal Omega webserver (49) and edited by open-source Jalview software version 2.11.1.3 (50). Amino acid sequence logos for the conserved motifs were generated using the WebLogo program (51). Phylogenetically informative sites were selected from the MUSCLE alignment result by using Gblocks in PhyloSuite v1.2.2 (49, 52). The phylogenetic tree was constructed using IQ-TREE in PhyloSuite with the maximum likelihood method of 1,000-fold bootstrap (52). Virus abbreviations and accession numbers are listed in Table S2.

### Data availability.

The GenBank accession numbers of WWPSRV-1, WWPSRV-2, AcPSRV-1, AcNSRV-1, AcNSRV-2, and LdNSRV-1 are MW864600, MW864601, MW864599, MW864602, MW864603, and MW864604, respectively. BioProject accession for the RNA-seq of three parasitoid wasps of the rice weevil *Sitophilus oryzae* is PRJNA719021.
