# Supplementary figures and images for: Diverse RNA Viruses Discovered in Three Parasitoid Wasps of the Rice Weevil Sitophilus oryzae
Source: mSphere. 2021 May 5;6(3):e00331-21. doi: 10.1128/mSphere.00331-21 (PMC8103988; doi:10.1128/mSphere.00331-21)

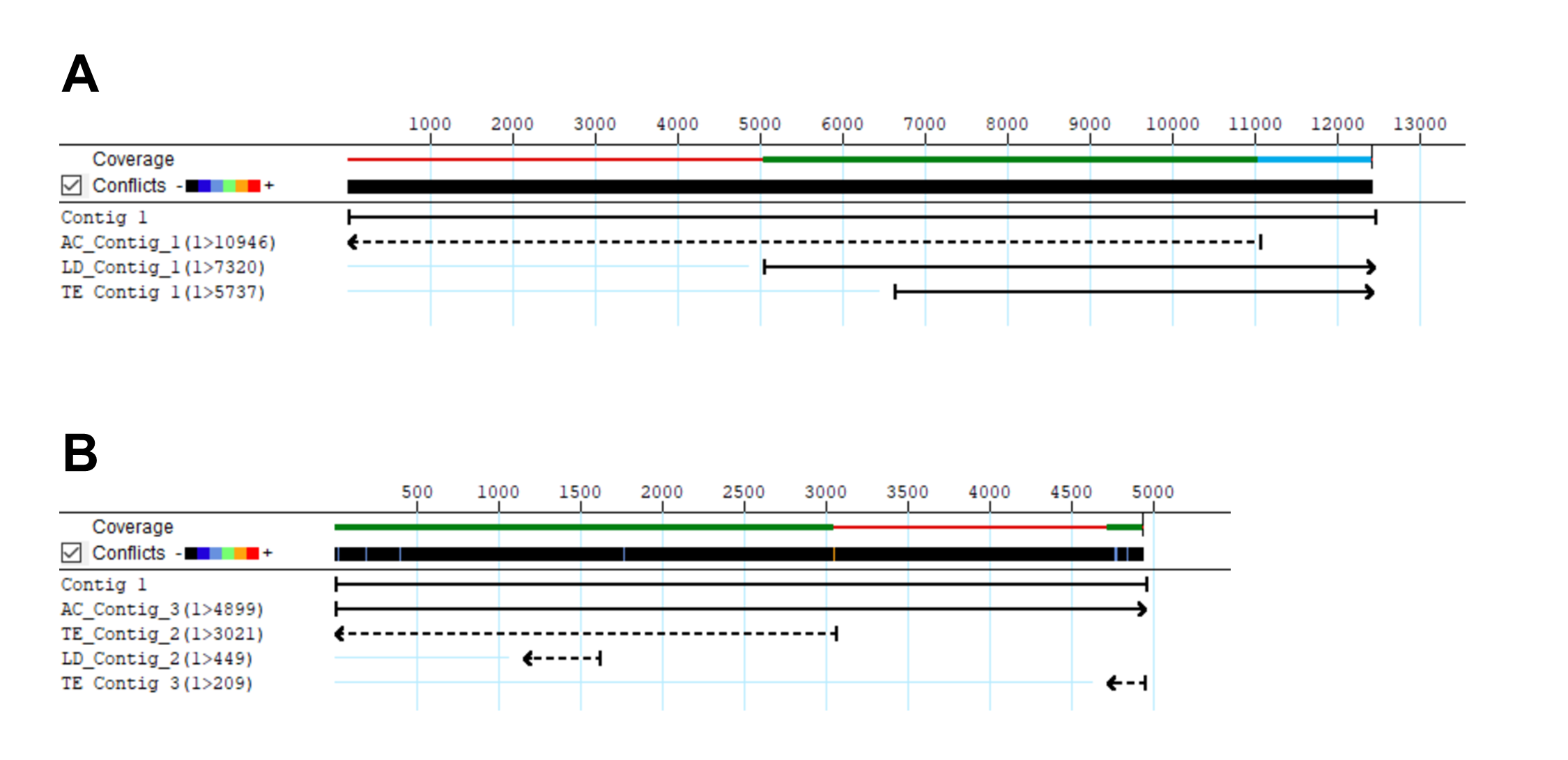

Supplement: FIG S1 [file mSphere.00331-21-sf001.tif]

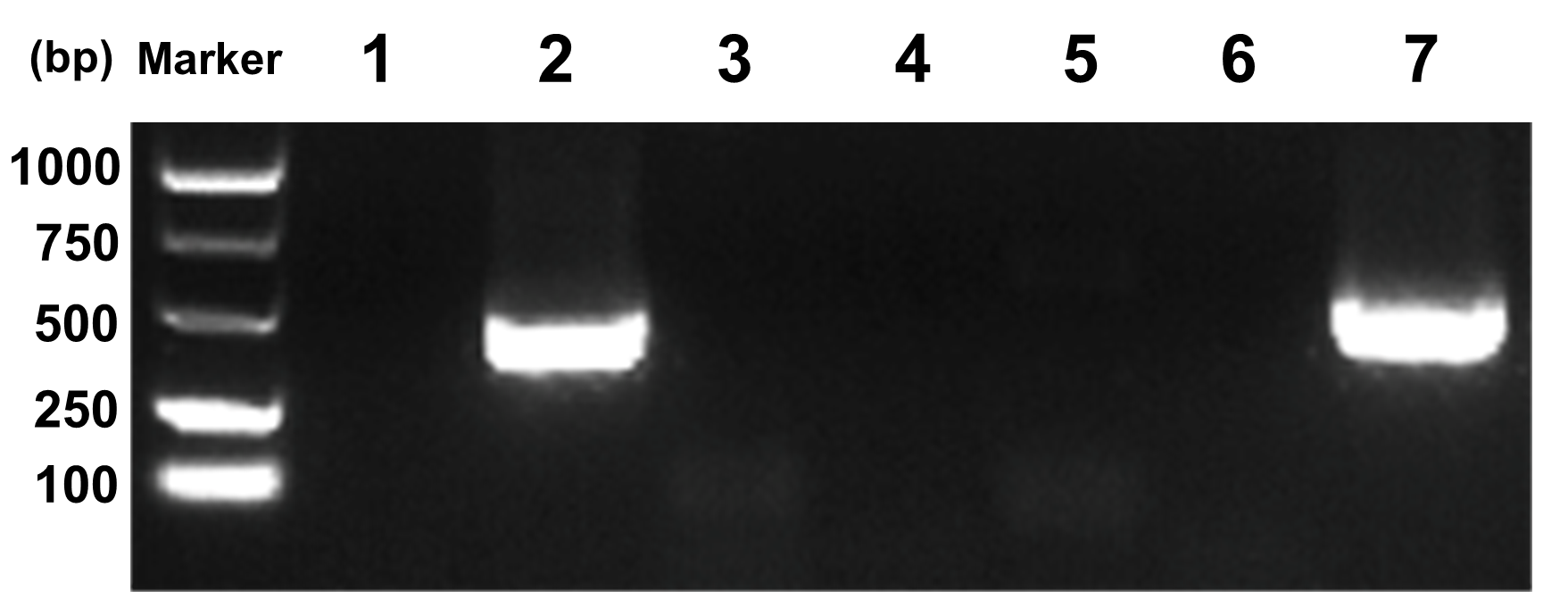

Supplement: FIG S2 [file mSphere.00331-21-sf002.tif]

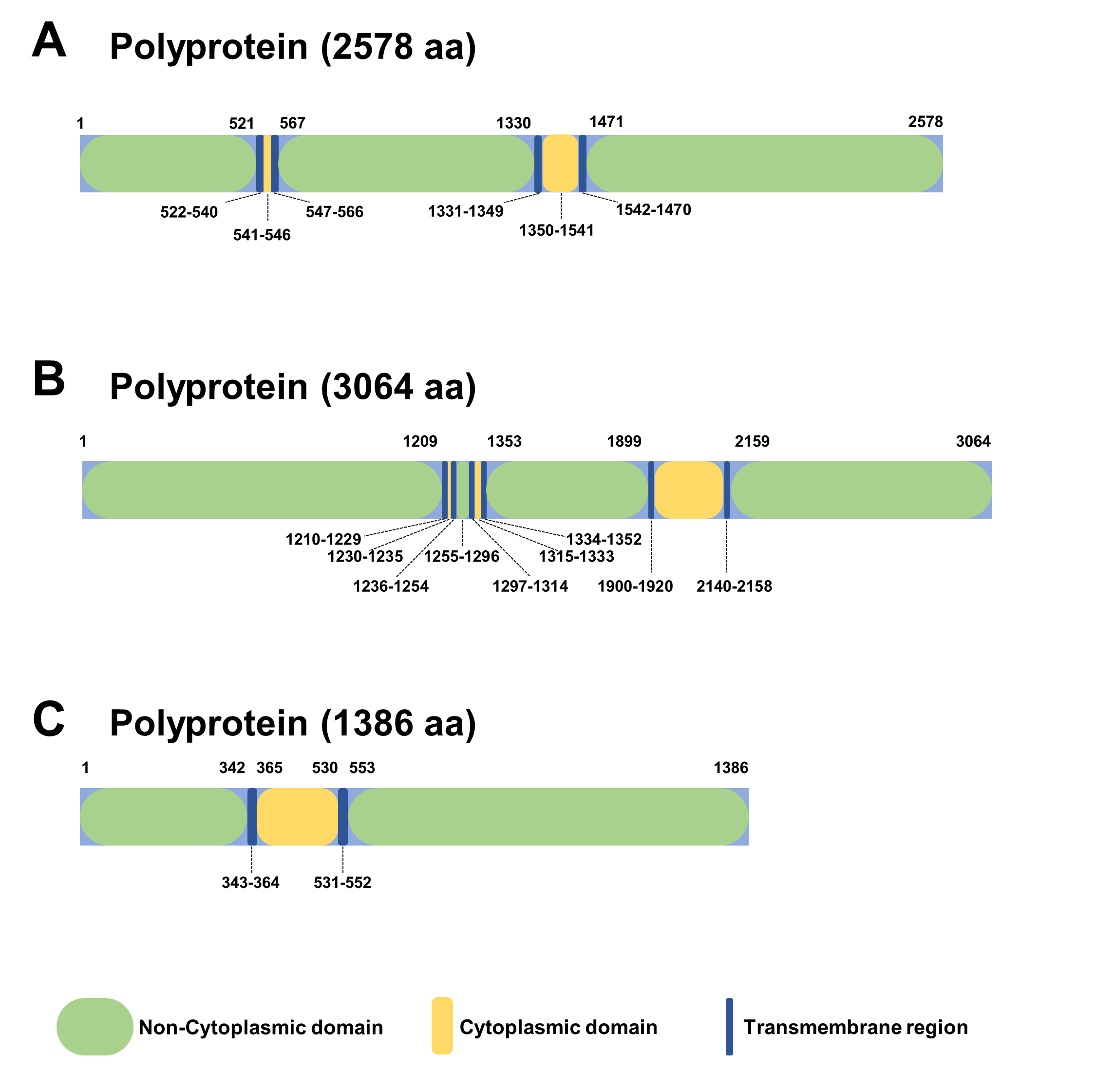

Supplement: FIG S3 [file mSphere.00331-21-sf003.tif]

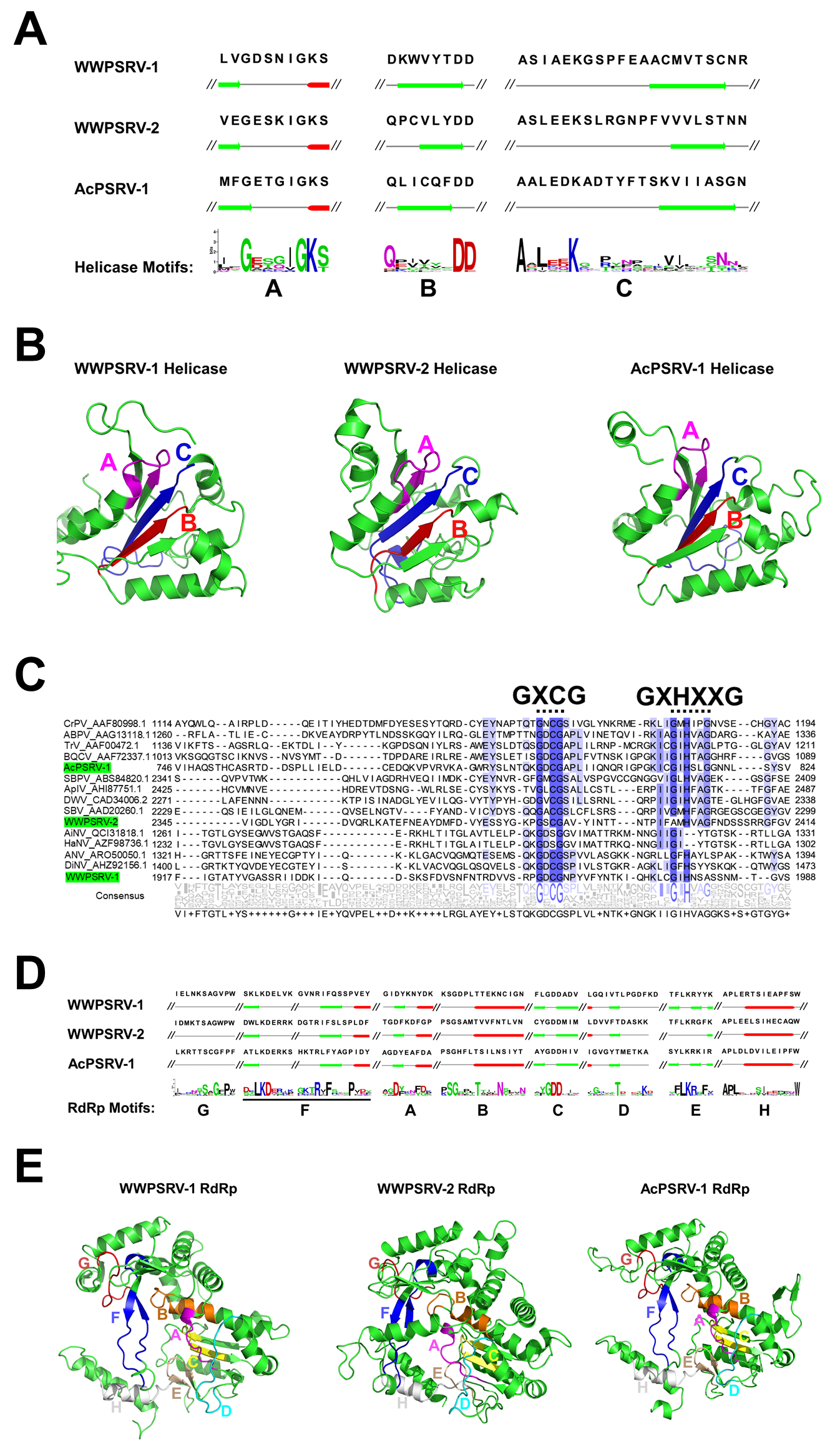

Supplement: FIG S4 [file mSphere.00331-21-sf004.tif]

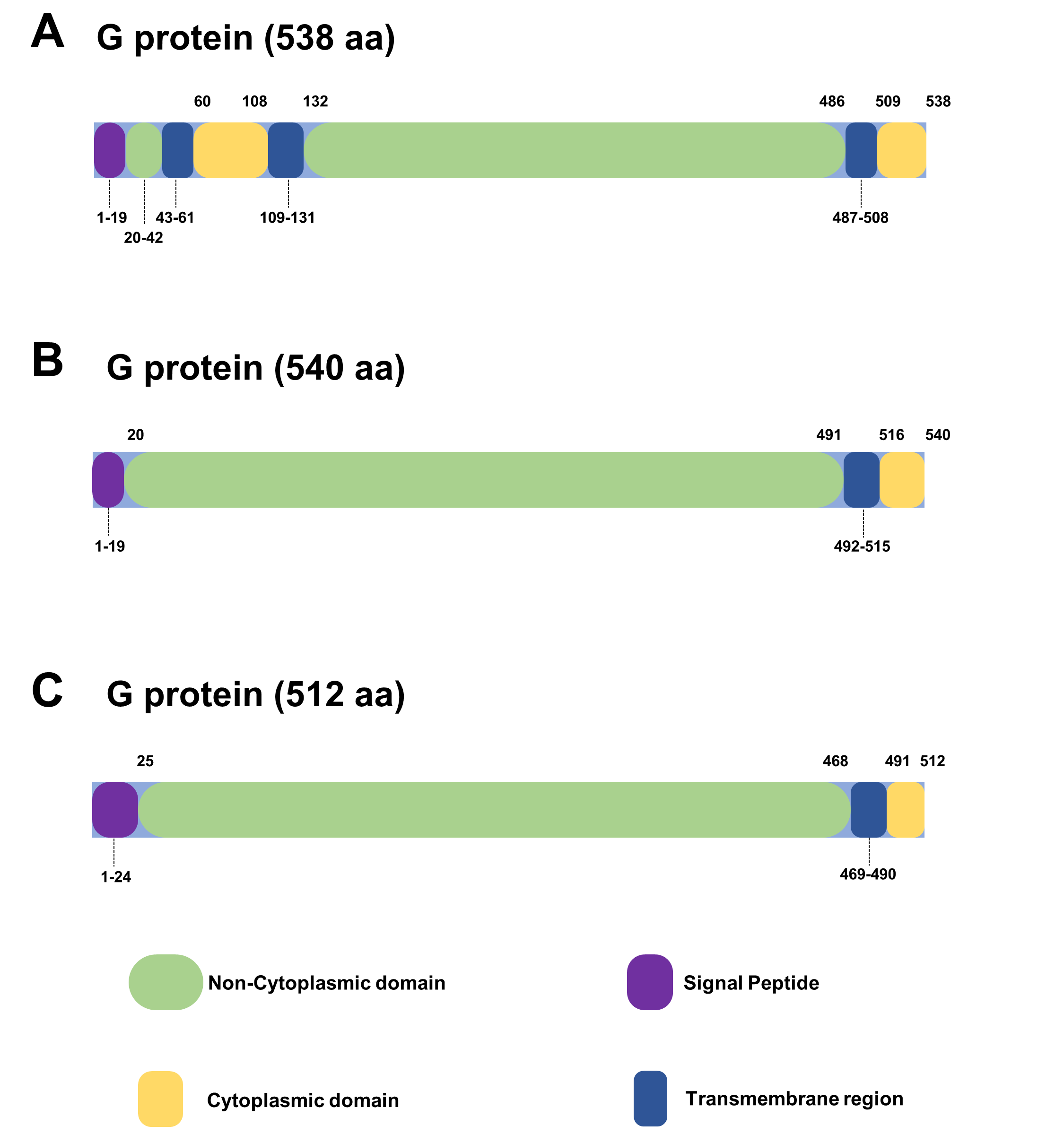

Supplement: FIG S5 [file mSphere.00331-21-sf005.tif]

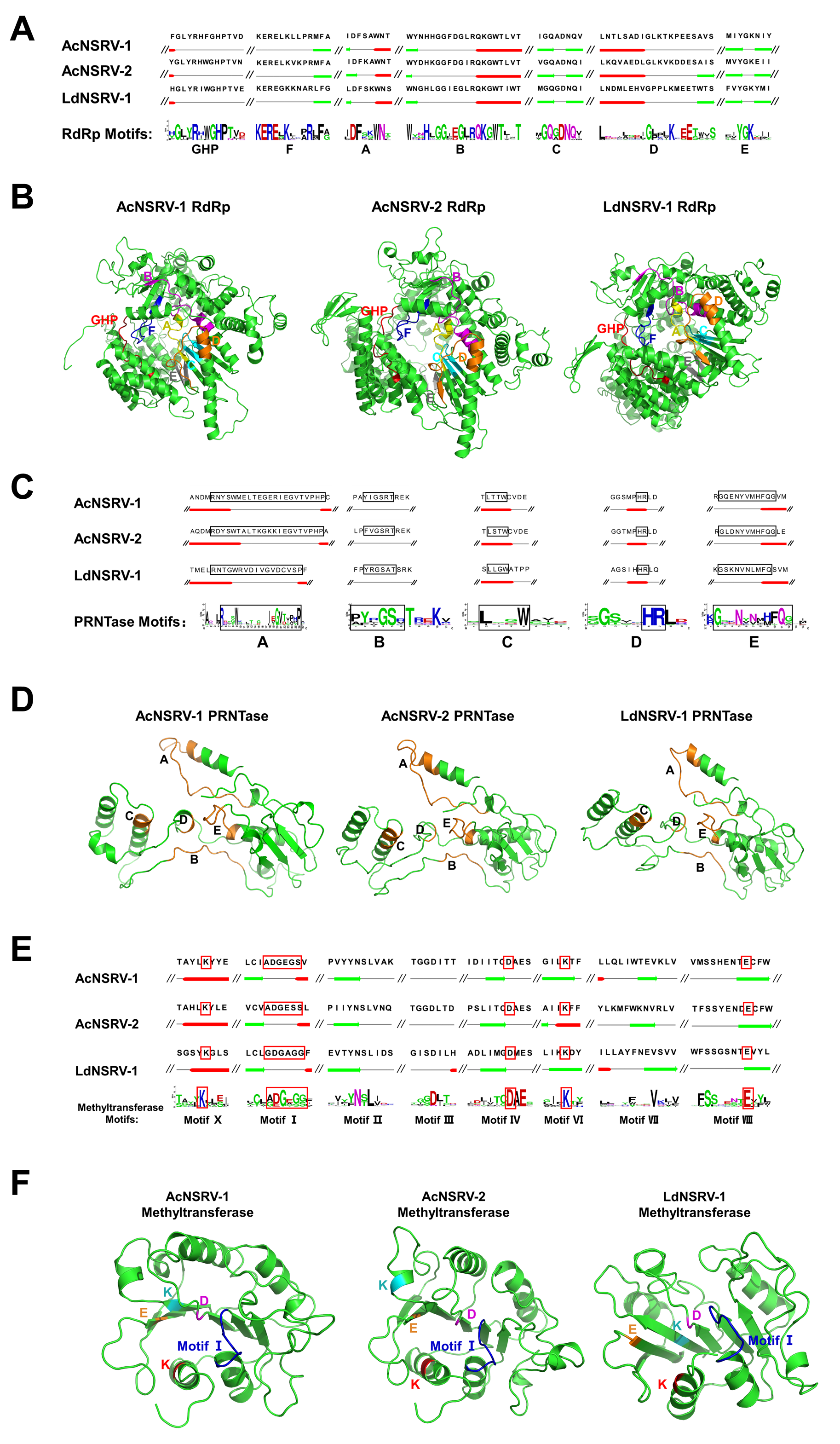

Supplement: FIG S6 [file mSphere.00331-21-sf006.tif]
